# Role of polysaccharides in food, digestion, and health

**DOI:** 10.1080/10408398.2014.939263

**Published:** 2015-04-28

**Authors:** A. Lovegrove, C. H. Edwards, I. De Noni, H. Patel, S. N. El, T. Grassby, C. Zielke, M. Ulmius, L. Nilsson, P. J. Butterworth, P. R Ellis, P. R. Shewry

**Affiliations:** ^a^ Department of Plant Biology and Crop Science, Rothamsted Research, Harpenden, Herts, United Kingdom; ^b^ King's College London, Diabetes and Nutritional Sciences Division, School of Agriculture, Policy and Development, London, United Kingdom; ^c^ Department of Food, Environmental and Nutritional Sciences, University of Milan, Milan, Italy; ^d^ Food Engineering Department, Nutrition Section, Ege University, Izmir, Turkey; ^e^ Food Colloids Group, Department of Food Engineering, Technology and Nutrition, Faculty of Engineering LTH, Lund University, Lund, Sweden; ^f^ Solve Research and Consultancy AB, Lund, Sweden; ^g^ Department of Agriculture, Reading University, Whiteknights, Reading, Berkshire, United Kingdom

**Keywords:** Starch, nonstarch polysaccharides, dietary fiber, food processing, health benefits

## Abstract

Polysaccharides derived from plant foods are major components of the human diet, with limited contributions of related components from fungal and algal sources. In particular, starch and other storage carbohydrates are the major sources of energy in all diets, while cell wall polysaccharides are the major components of dietary fiber. We review the role of these components in the human diet, including their structure and distribution, their modification during food processing and effects on functional properties, their behavior in the gastrointestinal tract, and their contribution to healthy diets.

## Introduction

Understanding the relationships between the compositions of raw food materials, the effects of processing on their structures and interactions, and their behavior in the gastrointestinal (GI) tract are crucial for elucidating the relationships between diet and health. Although plant foods provide a range of essential dietary components, they are particularly important as a source of dietary carbohydrates, providing almost all of the carbohydrate, and therefore much of the energy, in the adult diet. For example, Subar et al. (1998) list only plant sources as contributing to carbohydrate intake in the US adult diet, accounting for 60% or more of the energy intake. Plant carbohydrates can be divided into two classes, which have contrasting but important impacts on the diet and health of humans. The first is storage carbohydrates, particularly starch, but also oligosaccharides and sugars (which are not considered here). The second group is the cell wall polysaccharides, which are derived in our diet mainly from plants, but also from fungi and algae (either directly or added as ingredients).

Starch is the major storage carbohydrate in plants, and the major source of calories in many plant organs and foods. Its biophysical properties also have major impacts on food texture and other properties. Although available starch is readily digested in the small intestine, resistant starch (RS) and cell wall polysaccharides (or nonstarch polysaccharides, NSPs) are not digested, but are the major components of dietary fiber and are fermented by the colon microbiota to produce short chain fatty acids (SCFAs). NSPs provide a rigid structure surrounding plant cells and therefore affect the release and digestion of the cell contents. Table 1 provides a summary of the major types of plant-derived carbohydrate in the human diet.

**Table 1.** Major food sources and structure of carbohydrates present in the diet.

Class	Polymer	Structure	Major sources
Starch	Amylose Amylopectin	(1→4)-α-linked D-glucose (1→4)-α-linked D-glucose (1→6)-α-linked branches	Cereals, tubers, legumes, pulses
Glucose	CelluloseCalloseMixed linkage glucan Mixed linkage glucan	(1→4)-β-linked D-glucose (1→3)-β-linked D-glucose (1→3,1→4)-β-linked D-glucose (1→3,1→6)-β-linked D-glucose	Fruit, vegetables Cereal grains Seaweeds, yeast and other fungi
Hemicellulose	Xyloglucan Glucomannan	(1→4)-β-linked D-glucose (1→6)-α-linked D-xylose substitutions(1→4)-β-linked D-mannose (1→6)-α-linked D-glucose substitutions	Fruit, vegetables, tamarind
	GalactomannanGlucuronomannansGalactansArabinoxylanGlucoronarabinoxylans	(1→4)-β-linked D-mannose (1→6)-α-linked D-galactcose substitutions(1→2)-β-linked D-mannose and D-glucuronic acidD-galactose and L-arabinose substitutions (1→3)-β-galactose (1→4)-3,6-anhydro-α-D or L-galactose (1→4)-β-xylose(1→2)-α and (1→3)-α-L-arabinose and ferulyolated L-arabinose substitutions As arabinoxylans with D-glucuronic acid substitutions	Guar, locust, and carob beans Fungi, algae Seaweeds (carageenins, agar)Cereal grainCereal grain
Pectins	Homogalacturan (HG)(RGI) Rhamnogalacturan I (RGI)Rhamnogalacturan II (RG II)	Highly methyl esterified chains of (1→4)-α-D-galacturonic acidRepeated (1→4)-α-linked D-galacturonic acid (1→2)-α-D-rhamnose disaccharides. Substitutions of rhamnose with (1→4)-β-galactan, arabinan, arabinogalactan chains HG backbone with side chains containing several types of sugar linkage	Fruit and vegetablesFruit and vegetablesFruit and vegetablesFruit and vegetables
Oligosaccharides	Fructans Raffinose Stachyose	(1→2)-β-linked-D-fructoseD-galactose (1→6)-α-D-glucose (1→2)-β-D-fructoseGalactose (1→6)-α-raffinose	Chicory, Jerusalem artichoke, cerealsLegumes, vegetablesLegumes, vegetables

## Structure, occurrence, and properties of starch and nonstarch polysaccharides

### Structure and occurrence

Starch is a mixture of two glucose polymers: amylose, which comprises (1→4) α-linked chains of up to several thousand glucose units and amylopectin which is highly branched (with (1→6) α-linkages as well as (1→4) α-linkages) and may comprise over 100,000 glucose residues. Amylose is largely unbranched but may contain a few long branches (Takeda and Hizukuri, 1987), which occur more frequently in tuber starches than in cereal starches (Hizukuri, 1996; Hoover, 2001). Most starches consist of 20–30% amylose and 70–80% amylopectin, though mutations in the biosynthetic pathway or intentional manipulations via transgenic engineering can result in forms of starch with altered amylose:amylopectin ratios. Plant sources also exist with lower amylose:amylopectin ratio, for example, in some pseudocereals, such as amaranth and quinoa, with 8–12% amylose (Qian and Kuhn, 1999). However, due to the presence of low amounts of branches in amylose it is slightly misleading to strictly define the amylose:amylopectin ratio (Vilaplana et al., 2012).

Amylose and amylopectin are deposited within specialized plastids (called amyloplasts) in highly organized granules which vary in their abundances, sizes, and shapes between different species (Tester et al., 2004).

**Figure 1.**
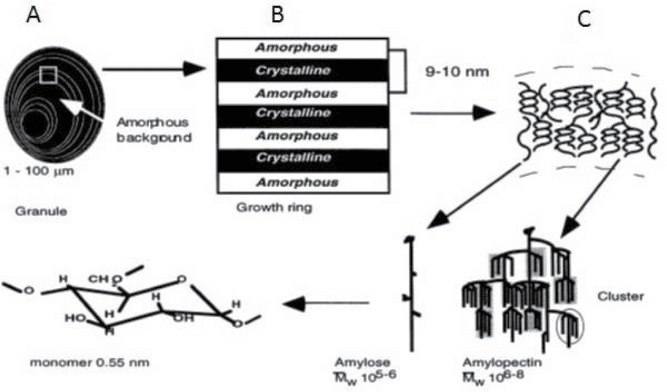
A schematic illustration of the organization of starch in a native granule. (A) Structure of a native starch granule showing alternating regions of amorphous and semicrystalline growth rings. (B) A semicrystalline growth ring showing the repeated layers of amorphous and crystalline regions. (C) Lamellar microstructure of the starch granule displaying the amylose chains in amorphous regions and the amylopectin helices in crystalline regions. Adapted from (Buléon et al., 1998).

Starch granules can vary in size and shape from <1 µm to >100 µm depending upon the source. Examination of the starch granules by microscopy with polarized light reveals a birefringence pattern with a characteristic “Maltese cross” indicative of a high degree of molecular orientation within the starch granule. The starch granules have a semicrystalline structure (alternating crystalline and amorphous regions) separated by amorphous growth rings (Fig. 1).

NSPs are made up of the hexose sugars glucose, galactose and mannose, the deoxy-hexoses rhamnose and fucose, glucuronic and galacturonic acids, and the pentose sugars arabinose and xylose (Bach-Knudsen, 2001). NSPs may be broadly grouped into classes (based on Waldron et al., 2003).

Cellulose is an unbranched chain comprising up to approximately 15,000 (1→4)-β-D-linked glucose units. The (1→4)-β-D-linked glucose chains associate by hydrogen bonding (both between and within strands) conferring strength to the cell wall. The linear polymers aggregate into either amorphous or crystalline regions. Cellulose is present in fungi, algae and higher plants, ranging from a few percent to 90% of the NSPs; the higher value being found in cotton fiber (Coffey et al., 2006). Cellulose is insoluble in water and indigestible to human enzymes, but fermented to varying degrees by microbes in the large intestine.

The term hemicellulose is sometimes used to refer to noncellulosic cell wall polysaccharides that are only solubilized by alkali treatment. Most cell walls contain hemicelluloses, but there is great variation in the exact content between species, tissues types, and cell wall layers.

Arabinoxylans are generally found in monocot cell walls, particularly grasses, and consist of a (1→4)-β-linked D-xylose unit backbone which is substituted with L-arabinose at either the 3 or the 2 and 3 positions. Further modifications of these chains may occur, including the feruloylation of monosubstituted arabinose units, which may lead to oxidative cross-linking of cell wall components. Arabinoxylans from wheat grain are of particular interest as they constitute the principle source of fiber in flour (and make up ˜70% of the cell wall polysaccharide) (Fincher and Stone, 1986). Water-soluble arabinoxylans have profound effects on processing properties (including bread making as discussed by Courtin and Delcour, 2002) owing to their water holding capacity and effect on viscosity. The water solubility is strongly related to the degree of branching (substitution), the more branched arabinoxylans are more soluble than less branched arabinoxylans (Izydorcryk and Biliadris, 1995). The solubility of arabinoxylan in wheat and other cereal grain may also be affected by feruloylation, which occurs at the 5 position of arabinose units. This substitution with ferulic acid allows the formation of cross-links, by oxidation of ferulate present on adjacent AX chains to give dehydrodimers (diferulates). Such cross-linked arabinoxylans may be important components of water-insoluble arabinoxylans, particularly in cereal brans. Substitution may also occur with *p*-coumaric acid, but at a lower frequency than with ferulic acid.

Xyloglucans consist of (1→4)-β-linked D-glucose units substituted with (1→6)-α-linked D-xylose, which may be further substituted (Fry, 2011). The best characterized xyloglucans are from tamarind seed (used commercially as gums), but xyloglucans are present in most land plant cell walls (Popper et al., 2011).

Glucomannans comprise glucose and mannose units with β–(1→4) linkages and may also have (1→6)-α-linked glucose substitutions.

Galactomannans are (1→4)-β-linked mannans substituted with (1→6)-α-linked galactose. Galactomannans are widely used in the food industry as thickeners and stabilizers; they are derived mainly from guar, locust, and carob beans (seeds). The glucuronomannans are (1→2)-β- linked mannose and glucuronic acid chains with substitutions of galactose and arabinose and are present in some edible fungi and alga. Galactan polysaccharides, (which can be sulfated) consisting of (1→3)-β-D galactose and (1→4)-3, 6-anhydro-α-D-galactose units, are found in numerous red seaweeds, and are used widely in the food industry as gels and thickeners. Carageenans and agars, which differ in whether the (1→4) anhydro galactose is the D- or L- form (Piculell, 2006), are also widely used.

Cereal mixed-linked β-glucans are glucose units linked (1→4)-β (as in cellulose) but interspersed with (1→3)-β-linkages. The (1→3)-β-linkages generally occur after three or four β-(1→4) linkages, but more extensive cellulose-like stretches of up to 20 (1→4)-β-linked residues have been reported in wheat bran (Li et al., 2006). The irregular linkage structure prevents the formation of an ordered crystalline structure, leading to the β-glucans being partially water soluble. Water-soluble mixed-linked β-glucans of barley (where they constitute ˜70% of endosperm cell wall polysaccharides (Fincher and Stone, 1986)) and oats are able to form viscous solutions and dispersions. In addition, b-glucans with (1→3)(1→6) linkages occur in fungi.

Pectic polysaccharides have an extremely diverse structure but share some common features, particularly the presence of galacturonic acid in the backbone of the polysaccharide. Three major pectic polysaccharides are recognized; homogalacturonan (HG), rhamnogalacturonan-I (RG I), and rhamnogalacturonan-II (RG II) (Willats et al., 2006). HG comprises (1→4)-α-linked D-galacturonic acid units with occasional rhamnose residues, up to 200 units long. RG I has a backbone of repeats of the disaccharide (1→4)-α-D-galacturonic acid (1→2)-α-L-rhamnose (up to approximately 10 units long; Thibault et al., 1993). The rhamnose residues may be substituted with (1→4)-β-galactan, branched arabinan, and/or arabinogalactan side chains. The nonbranched HG regions are commonly referred to as “smooth” while the branched regions are referred to as “hairy.” RG II has a HG backbone decorated with side branches (designated A–D), which consist of 12 different sugars and 20 different linkages (Mohnen, 2008). RG II is a very minor component of plant cell walls and has an extremely complex chemical structure including some very rare sugars (Voragen et al., 1995). The different pectic polysaccharides are not separate molecules, but consist of covalently linked domains (Harholt et al., 2010). Similarly to arabinoxylan, ferulylation of arabinose in pectin may also occur, as for instance, in sugar beet pectin. Another important feature in the chemical structure of the pectic polysaccharides is the presence of methyl esters at the carboxylic groups of the galacturonic acid as well as acetylated hydroxyl groups. The extent to which these modifications occur has a large influence on the aqueous solubility of the polysaccharide as well as its solution properties. If more than 50% of the carboxyl groups are methylated, the pectin is referred to as high methoxyl (HM) pectin and if less than 50% are methylated then the pectin is referred to as low methoxyl (LM) pectin. Pectins make up around 35% of primary cell walls of dicotyledonous and nongraminaceous monocots (Mohnen, 2008; Willats et al., 2006; Carpita, 1996).

The types of pectin, as with the other NSPs, depend upon botanical and tissue origin and even the developmental stage and specific cell type within the plant. Like many of the polysaccharides described here, they are used in the food industry as gelling and thickening agents.

Oligosaccharides may also be present in plant tissues, notably fructans, mainly consisting of ((1→2)-β-linked oligomers of fructose such as inulin), but also some glucose, which are present in many grass species and 15% of flowering plants (Hendry, 1993). Some of the most commonly consumed fructan-containing plants are cereals, garlic, onion, leek, chicory, and Jerusalem artichoke. Raffinose (a trisaccharide of galactose, fructose, and glucose) is present in vegetables, legumes, and cereals and stachyose (two galactose, one glucose, and one fructose) is found in legumes and vegetables.

### Solution and dispersion behavior

The solubility of polymers depends on several different factors and molecular properties, with contributions from monomer-solvent interactions as well as entropic contributions. The latter, in the case of polymers, is dominated by the conformational entropy (i.e., the number of degrees of freedom at every monomer-monomer bond in the polymer chain) rather than the entropy of mixing.

NSP (dietary fiber) is traditionally divided into two major groups as being either insoluble or soluble in aqueous solution (AOAC, 2007; AACC, 2010). This division has many practical advantages. However, it has limitations which are important and often neglected when discussing and trying to understand the functionality of NSP. In particular, when detailed analysis is performed it can sometimes be difficult to define NSPs as either soluble or insoluble. Furthermore, different degrees of solubility may exist over a given molar mass population of an NSP (Håkansson et al., 2012).

A first general requirement for polymer solubility is that the monomers interact favorably with the solvent molecules (in this case water). Hence, the monomers need to have polar properties which enable them to form hydrogen bonds with the surrounding water molecules. The monomers in polysaccharides are monosaccharides which typically display high water solubility. Several NSPs also contain charged monosaccharides (as in for instance carrageenan or, to varying degrees, pectin) which can have a strong positive influence on the solubility of NSPs. For instance, demethoxylation of pectin (resulting in carboxylic groups) increases the solubility considerably (Voragen et al., 1995).

Other properties may also influence the solubility of polysaccharides. Many polysaccharides, although considered water soluble, have a rather poor solubility in water and tend to aggregate and phase separate over time. Thus, aqueous polysaccharide solubility is often of an apparent nature and is not a true solubility in a thermodynamic sense. The implication of the apparent solubility is that kinetic aspects become important in polysaccharide solutions and dispersions. Time becomes an important factor as solutions may not be stable over time. The instability of a given solution does not necessarily involve macroscopic phase separation, but may cause aggregation of polysaccharides into supramolecular aggregates, which may still be sufficiently small in relation to the wavelength of incoming light to appear as clear “solutions.” Nevertheless, these changes may influence the properties and functionality of the polysaccharides.

Similarly, kinetics may play a large role in the dissolution of polysaccharides, with the dissolution of polysaccharides being very slow due to the properties of the matrix from which they have to be dissolved. Thus, polysaccharides may appear as insoluble or having low solubility depending on the time scale on which observations are made. In most cases, with the exception of some modified celluloses, solubility, and dissolution rate of food polysaccharides increases with increasing temperature.

When considering the solution behavior of polysaccharides, it is important to note that in most cases they occur in a food matrix which is a mixture of macromolecules. The solubility and mixing of the different macromolecules will then be dependent on the balance of the different interactions present. For example, both macromolecules may display high solubility and also repulsive interactions (incompatibility) resulting in segregative phase separation (Quiroga and Bergenståhl, 2008). However, both macromolecules may have high solubility and either lack, or have only weak interactions resulting in miscibility, or have strong attractive interactions resulting in association (coacervation). The resulting aggregates may either behave as soluble complexes (Weinbreck et al., 2003) or become insoluble resulting in macroscopic phase separation (Magnusson and Nilsson, 2011).

One of the most important functional properties of dissolved polysaccharides, both in relation to formulation functionality and health related functionality is the ability to thicken solutions and to form gels. The ability to increase the viscosity of solutions depends on the hydrodynamic volume of the polysaccharide with a large hydrodynamic volume resulting in increased viscosity at low concentration. From this, it follows that high solubility (i.e., good interaction with the solvent resulting in an expansion of the polysaccharide and larger hydrodynamic volume) is beneficial for the thickening ability. The thickening properties may be enhanced further if associative interactions are present in the polysaccharide, as for example, in some modified starches and celluloses.

The ability to form gels is dependent on a relatively high solubility of the polysaccharide, in order for the gel structure to be able to hold water, and the formation of a continuous network in the solution. Thus, some interaction between the polysaccharides leading to association is necessary. This association can be mediated either through the hydrophobic effect, through partial local crystallization, through calcium bridges or through the formation of double and triple helices between molecules.

It should also be noted that dispersed insoluble polysaccharides may also give rise to increases in viscosity and the formation of gels (Castro et al., 2012, 2013). However, in this case, the behavior is that of a particle suspension, i.e., the increase in viscosity is dependent on particle–particle interactions and gel formation occurs at relatively high volume fractions of the insoluble polysaccharides.

## Effects of processing on structure and composition

### Mechanical fractionation

Modifications of some properties of starch and NSPs may occur at the initial stage of mechanical processing. For example, the dehulling and milling of cereal grains and peeling and chopping of potatoes cause physical damage to a proportion of starch granules, resulting in loss of crystalline structure (Donald, 2004). Damaged starch possesses a water absorption capacity ten times greater than native starch and it is more prone to gelatinization with implications for end-use properties and digestion. Milling also disrupts cell wall structure and affects particle size. Other factors such as mill type and wheat variety can result in more or less protein or starch present in the flour fraction and the distribution of more or less of the soluble or insoluble NSPs into the various milling fractions (and hence food products) (Campbell et al., 2007).

### Heating (hydrothermal) and gelatinization

Many food processes involve heating (cooking) and/or cooling under variable moisture conditions, which cause structural changes at the food, granular, and molecular levels. Different processing conditions therefore have different effects on starch structure and accessibility, with implications for digestibility.

**Figure 2.**
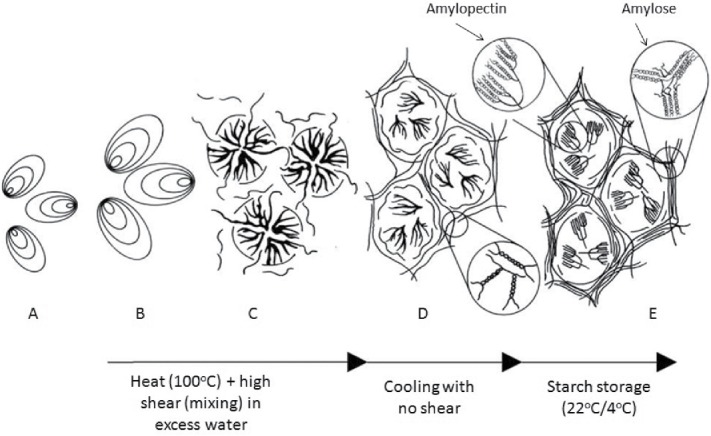
Effects of processing on starch granules. (A) Intact native starch granule. (B) Heat treatment in excess water under high shear conditions results in granular swelling. (C) Granule disruption occurs during starch gelatinization with linear amylose chains leaching out of the granule. (D) Upon cooling, amylose chains aggregate together to form an ordered gel network. (E) Recrystallization of amylopectin and amylose chains occurs upon storage of gelatinized starch. Schematic representation adapted from (Goesaert et al., 2005).

Heating native starch (50–100°C) in excess water results in gelatinization. During this process, the semicrystalline starch granule becomes completely disrupted. Hydrogen bonds that hold the double helical structure of α-glucan chains (the amylopectin fraction) together are broken, resulting in a greater proportion of amorphous starch material (Dona et al., 2010). Thus hydrothermal processing changes the morphology of starch granules, from an ordered to disordered structure (starch gelatinization is shown in Fig. 2). Starch gelatinization requires both heat and moisture, and is most rapid when starch is heated in excess moisture (>70 %) between 50 and 100°C (Roder et al., 2009).

Starches from different botanical sources, however, differ in their gelatinization behavior, and not all starch may be completely gelatinized during hydrothermal processing treatments. For instance, gelatinization of high amylose starches may require temperatures in excess of 120°C, which is considerably higher than normal (Sievert et al., 1990; Jacobs and Delcour, 1998; Haralampu, 2000). The presence of a resilient food matrix (e.g., cell wall structures or a protein network) may also restrict gelatinization of starch, by limiting water, heat transfer, or space for granular swelling, despite prolonged exposure to hydrothermal conditions (Wűrsch et al., 1986; Champagne et al., 1990).

Cooking a starch suspension results in rheological properties that are of value for foods. In general, starches derived from tuberous crops (e.g., potato or tapioca from cassava) tend to swell and thicken at lower temperatures than cereal grain starches. Cereal starches (e.g., maize and wheat) break down more slowly with prolonged cooking. High amylose cereal starches are more resistant to swelling and behave like tuber starches during thermal treatments, with amylose leaching to a greater extent than amylopectin. Also, the concentration of starch when cooked affects its swelling properties. Above certain concentrations, the swollen starch granules entrap all available water and the aqueous phase will not separate from them. Cooked granules, in contrast to unswollen ones, can be disrupted by shearing.

### Thermal degradation

The chemical structure of starches can be dramatically altered by heat treatments such as baking, toasting, roasting, and frying. These may result in starch degradation (decomposition) by breaking the glycosidic bonds within the polysaccharide chains. However, the thermal decomposition of starches from different botanical origins showed no significant relationship between microstructure (crystallinity, granule size) and the thermal degradation process (Guinesi et al., 2006). Dehydrated oligomers of glucose and individual molecules of dehydrated glucose are the predominant products of thermal decomposition of pure starch in model systems and in toasted bread (Golon et al., 2013). Sugars and oligomers released by thermal degradation as well as simple sugars resulting from amylolytic activity on (damaged) starch may be involved in the Maillard reaction during the above heat treatments (De Noni and Pagani, 2010). McDougall et al. (1996) noted that cooking alters the physical and chemical properties of NSP. Thermal degradation and solubilization of cell wall polymers may occur and the cells may separate or fracture, potentially increasing the accessibility and surface area. Cooking can also result in modification of NSP by the Maillard reaction (Pérez-Jiménez, 2014).

Pectin, being a relatively process-sensitive NSP, can undergo depolymerization upon heating, at neutral pH, at relatively intermediate heating temperatures (≥50°C) and times (Albersheim et al., 1960). Under heating at neutral pH and above, chemical β-elimination (Kiss, 1974) and demethylation (Renard and Thibault, 1996) occurs causing depolymerization while at pH4.5 acid hydrolysis dominates the depolymerization Furthermore, it has been shown that heating changes the conformational properties of pectin in solution which may also have an impact on pectin functionality (Diaz et al., 2007; Shpigelman et al.,2014).

Boiling of β-glucan solutions/suspensions results mainly in dissolution of larger aggregates while when heating to higher temperatures depolymerization may also occur (Ulmius et al., 2012a; Hakansson et al., 2012). Similarly, β-glucan in food products that are heat treated at higher temperatures (<100°C) have been shown to become depolymerized as a result of the processing which was also interpreted to influence their beneficial health effects (Regand et al., 2009; Tosh et al., 2010).

It should be noted that purely thermal effects on the depolymerization and degradation of NSPs can be difficult to distinguish from enzymatic degradation. This is especially true for NSPs present in their native food matrix and, thus, in the presence of depolymerizing enzymes such as in the case for β-glucan in bread (Regand et al., 2009) and pectin in vegetable tissue (Castro et al., 2013).

### Cooling and retrogradation

When starch gelatinization is followed by cooling, structural changes occur to the α-glucan chains in that the initial predominantly amorphous state begins to reform a more ordered or crystalline structure. This process is termed retrogradation (Htoon et al., 2009) and is affected by the presence of sugars or other hydroxyl-containing molecules (Gudmundsson and Eliasson, 1990). (The rate of retrogradation is increased at lower temperatures with a slow rate of cooling favoring a more organized association of amylose molecules (and hence stronger gels).)

The chemical properties of starch and the cooling conditions play a major role in predisposing starch to thicken or form a gel. This phenomenon involves the retrogradation (recrystallization) of the glucan polymer chains by hydrogen bonds forming starch crystallites which are less favorable to amylolysis (Tester et al., 1998). The recrystallization of amylopectin chains has been noted to be much slower than amylose chains (Ring et al., 1987; Miles et al., 1985). The content and molar mass of amylose also affect the strength and opaqueness of cooled gels.

The degree of crystallinity in retrograded starch has been extensively studied using wheat bread as an economically important example. When the retrogradation of starch occurs, water migrates toward the crust, resulting in the crust having a higher moisture content than the crumb and in staling and hardening of the bread (Eliasson et al., 2013). Although the molecular structure of retrograded starch does not return to the original native starch structure, the recrystallization step does result in fewer available α-glucan chains for α-amylase to bind to, thereby reducing starch digestibility.

### Extrusion and other treatments

Extrusion processing pushes material through an orifice or die using a screw, during which heat, high pressure, and shear forces are applied. Mixing, particle size reduction, melting, texturizing, and browning may also occur. However, mechanical energy input is the primary mechanism for cooking (Whalen et al., 2000). The result of extrusion may be partial gelatinization or an expanded porous structure. The overall effect on starch depends on the physical and chemical features of the raw materials and the processing parameters, mainly moisture, time/temperature, and shear stress. After extrusion, almost no native tissue or granular starches are retained (refer to Fig. 2).

Heat-moisture treatment (HMT and annealing (ANN) involve the treatment of starch under specific time/temperature and moisture conditions and modify the polysaccharide chain conformation and helicity without destroying the granular morphology or changing the birefringence of the granules (Mishra and Monro, 2012; Hoover, 2010). The amylose:amylopectin ratio and the arrangement of the starch chains within the amorphous and crystalline domains of the native granules from different sources influence the susceptibility of starch to HMT and ANN.

High hydrostatic pressure (HHP) treatment up to 150–250 MPa decreases the gelatinization temperature as a result of alteration in granule structure (Blaszczak et al., 2005). HHP-gelatinization of a starch suspension depends on the treatment pressure, starch concentration and origin, temperature, and time (Liu et al., 2010; Katopo et al., 2002). The extent of gelatinization achieved during the pressure treatment determines the rheological properties of the HHP-treated product (Stolt et al., 1999).

### Effect of processing on NSPs

The behavior of NSPs in processing will vary between the precise polymer type and the species. Soluble NSPs affect the viscosity of a solution, depending on the solubility, concentration, structure, molar mass, and possibly the ability to form aggregates (Kumar et al., 2012; Gómez et al., 1997). NSPs that contain charged groups (such as pectic polysaccharides) interact more favorably with polar solvents, such as water, which can thus increase their solubility. Changes in pH affect the charge on the NSPs, and can cause a decrease in solubility and trigger the formation of networks and gels; conversely it can also cause partial or complete depolymerization. Side-chains and structural irregularities can also lead to higher solubility and hence, less network formation. Milling to smaller particle sizes can promote dissolution of NSPs due to the increase in surface area and by eliminating potential physical barriers, to allow fluid penetration (Wang et al., 2006). A high molar mass of NSPs is typically associated with increased viscosity, but also with decreased solubility (Wang et al., 2003). Hence, a trade-off may exist between these characteristics. A high concentration of NSP typically increases the viscosity. Low molar mass can, on the other hand, increase the degree of aggregation due to higher diffusion rates (Li et al., 2011).

The NSP polymer structure and molar mass may be affected by processing including typical food processes. High temperatures have been demonstrated to degrade the individual polymers of β-glucans leading to lower molar mass and reduced viscosity (Regand et al., 2009; Tosh et al., 2010). However, heat treatment at 100°C (boiling) only reduced large polymer aggregates and resulted in increased solubility without affecting the molar mass (Ulmius et al., 2012a; Beer et al., 1997). Hence, boiling may impart viscosity *enhancing* effects.

Bread baking not only increases the solubility of arabinoxylans, but also results in depolymerization, due to the action of enzymes in the flour (Courtin and Delcour, 2002). Frozen storage of food products containing β-glucans are reported to result in decreased solubility with no change in molar mass (Beer et al., 1997). Products, such as oat bran muffins, exposed to freeze-thaw cycles have been reported to exhibit lower β-glucan solubility, explained as an effect of cryogelation (Lan-Pidhainy et al., 2007). Similarly, solutions of β-glucans exposed to freeze-thaw cycles results in aggregate formation and eventually in macroscopic phase separation due to loss of solubility (Ulmius et al., 2012a).

### Effects of Interactions of starch with other components

The complexation of lipids with amylose may occur during extrusion cooking resulting in effects on functional properties such as viscosity and reduced starch digestibility of extruded foods (Altan et al., 2009). Proteins and other components (such as NSP) affect both the degree of gelatinization during food processing and the digestion of starch (Willett et al., 1994; Giacco et al., 2001).

Proteinaceous enzyme inhibitors (e.g., albumin and gliadin inhibitors) and a range of polyphenolic compounds have been shown to limit starch digestion (Rehman, 2005; Wűrsch et al., 1986; Singh et al., 1982). However, the potential of inhibitory proteins to severely restrict starch digestion in vivo is likely to be limited, because they are generally thermally unstable and susceptible to gastric proteases (e.g., pepsin) (Buonocore, 1977; Wűrsch et al., 1986; Frias, 2000).

### Carbohydrate modification

Starches can be modified to alter their properties. For example, cross-linking of starch gives more stable gels, which are not disrupted by (over)cooking, changes in pH, and shear during processing. Depending on the type and degree of cross-linking, the derived modified starch becomes less affected by acid and sugar, and more resistant to acid, heat, and shear than native starch (Jyothi et al., 2006).

Other modifications include partial hydrolysis (by heating in the presence of acid or alkali), bleaching, or oxidation to generate carboxyl groups and formation of esters and ethers. Chemical modifications introduce functional groups into the starch molecule, resulting in altered chemical and physical properties (Abbas et al., 2010). Starch esters (acetate, phosphate, and octylsuccinylated) and ethers (carboxy methyl and hydroxyl propyl) retard retrogradation, increase water binding capacity, lower gelation temperature, and impart emulsifying properties. Many modified starches are both cross-linked and substituted, and the level of each of these processes is varied to adjust functionality depending on the end use (Abbas et al., 2010). Some modifications of starches can alter the susceptibility to enzymic hydrolysis and/or their gelatinization behavior (Han and BeMiller, 2007; Chung et al., 2008; Hwang et al., 2009).

Many of the above modifications apply also to NSPs, for example cellulose, pectins, carrageenans, and alginates are all used and modified by the food industry to improve their functionality. Thus, their uses can be extended or improved (Yalpani, 1999; Geresh et al., 2002; Wang et al., 2004; Yuen et al., 2009). The food industry has found the ether derivatives of cellulose useful to confer specific rheological, emulsification and, foam stability properties to foods, and also control ice crystal growth and formation, and water-binding capacity (Coffey et al., 2006).

However, cellulose and its derivatives and the NSPs described above remain indigestible by human digestive enzymes. Thus, they constitute dietary fiber, along with other undigested polysaccharides such as RS (see below), which are fermented in the colon by bacterial enzymes (Waldron et al., 2003).

### Digestibility of polysaccharides

Starch digestibility is influenced by a number of factors. At the molecular level, the ratio of amylose to amylopectin influences starch digestibility. The amylose chains form a single helical structure which packs predominantly within the amorphous regions of the starch granule. These amorphous regions are more susceptible to amylase hydrolysis (Buléon et al., 1998). Amylopectin, however, is less susceptible to amylolysis due to the tight packing of double helical chains in the semi-crystalline regions of the granule (Wang et al., 1998). Thus, in the native (uncooked) state, higher digestibility is observed for high amylose starches, which have a low degree of crystallinity (Bogracheva et al., 1995; Tahir et al., 2010a).

Once gelatinized, however, starch structure becomes more amorphous, and the α-glucan chains in the amylopectin-rich regions become exposed, thereby the ratio of amylose and amylopectin has little effect on overall digestibility in gelatinized starches (Tahir et al., 2010b). If the starches are then subjected to cooling, amylose retrogradation occurs, resulting in reduced digestibility of this component. The amylopectin remains digestible for a longer time because of the slower retrogradation. Therefore, the processing techniques outlined above influence this structure and thereby have major effects on digestibility.

**Figure 3.**
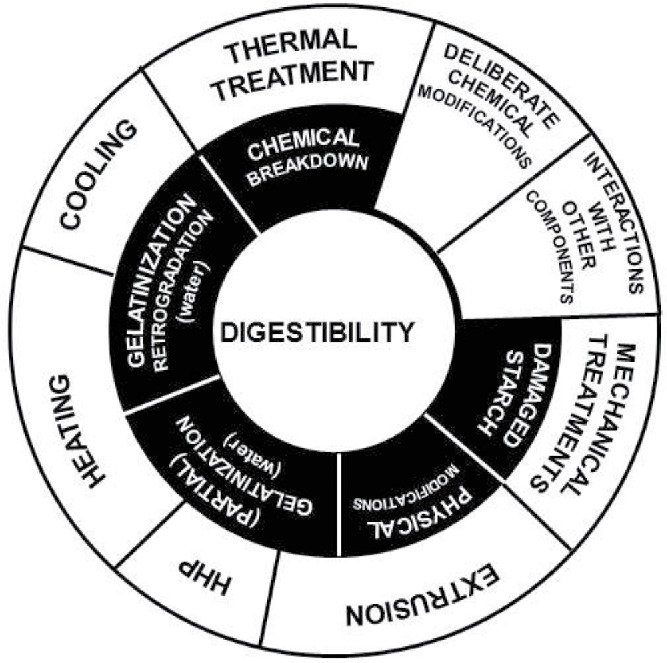
Food processes targeting starch digestibility. (HHP: high hydrostatic pressure).

Food macrostructure clearly has major effects on starch digestibility and postprandial glycemia and insulinemia (Bjorck et al., 1994; Parada and Aguilera, 2011). Food particle size, for example, is an important predictor of starch bioaccessibility, as the fractured particle surface is exposed to enzymes, whereas diffusion is required for enzymes to access starch in the underlying cell layers (Al-Rabadi et al., 2009). In many foods, the plant cell wall constitutes a diffusive barrier. However, the permeability of various cell walls to amylase has yet to be determined. Interestingly, micrographs of intact starch-containing cells recovered at the terminal ileum indicate that leguminous cell walls may, protect intracellular nutrients (e.g., starch) from digestive enzymes (Noah et al., 1998). Overall, the physico-chemical properties of the material is likely to influence the structural and chemical changes that occur during processing and digestive transit, and thereby has implications for digestibility. Fig. 3 illustrates the food processes that effect starch digestibility.

Cell wall polysaccharides range from the completely insoluble cellulose to soluble forms of β-glucan and arabinoxylan. As already mentioned, β-glucan and arabinoxylan are not digested by mammalian enzymes but fermented by bacterial enzymes present in the colon, and it is not known whether the solubility affects the rate of fermentation.

## Physiological aspects

Food structure affects metabolic responses to starch in cereal and legume products and any process disrupting the tissue or cell structure will alter these responses. Encapsulation of the cellular contents by plant cell walls has significant impacts on the rate and extent of nutrient release during digestion and consequently on the post prandial responses (Ellis et al., 2004; Mandalari et al., 2008; Berry et al., 2008). A high amylose content, or a high proportion of soluble NSP (high viscosity) has been reported to reduce glucose and insulin responses (Bjork et al., 1994).

### The mouth

The mouth is a complex environment. Food is broken down by chewing and mixed with saliva and enzymes. Some components are dissolved, there is a temperature change and the food is processed into a bolus and swallowed (Norton et al., 2006).

Starch digestion commences in the oral phase, as food is exposed to saliva consisting of electrolytes, lubricants, antimicrobial compounds and various enzymes, such as amylase, lipase, ribonucleases, and proteases (Varga, 2012).

Mechanical mastication facilitates starch digestion by mixing food with saliva and may also reduce the particle size, effectively increasing the availability of carbohydrate as the food matrix becomes disrupted

The physico-chemical modifications that occur during oral processing are of importance for carbohydrate digestion. However, it is not clear whether the contribution of the oral phase results predominantly from digestion by salivary enzymes, or from particle size reduction. Saliva also reduces the viscosity of food, thereby increasing enzyme access (Evans et al., 1986), but the extent to which salivary α-amylase contributes to total starch breakdown is controversial. Notably, the duration of starch exposure to salivary amylase is very brief (i.e., seconds-minutes) compared to the exposure to pancreatic α-amylase (i.e., hours) (Dahlqvist et al., 1961; Butterworth et al., 2011; Hoebler et al., 1998; Woolnough et al., 2008). Mastication can produce a range of particle size distributions depending on the nature of the ingested food. Some foods are masticated to achieve a small particle size whereas others, such as spaghetti, are swallowed as 5–12.5-mm-long sections (Hoebler et al., 2000). Mastication would be expected to increase the extent of starch digestion and thereby glycemia, by increasing the surface area for enzyme action.

The behavior of plant cell walls (and the NSPs that comprise them) during ingestion and digestion can have marked effects on the textural and nutritional properties of foods. Depending on the composition of the walls and the processes that have been applied to them, chewing may induce cell rupture or cell separation. Cell separation occurs when the individual cell walls are stronger than the adhesive force between them, whereas if the adhesive force is stronger, cell rupture occurs. In general, when the cell walls are rich in pectin, cell wall separation will occur after hydrothermal processing, most likely due to calcium ions in the middle lamella being solubilized by the water and/or degradation of the pectin chains. Separation of the cells allows the intracellular nutrients to remain encapsulated in cell walls. If the cell walls are not permeable to digestive enzymes, then minimal digestion of the cell contents will occur until either the cell wall is breached by bacterial degradation in the colon or the digestive conditions change the permeability of the cell wall. In unprocessed foods, cell wall rupture is much more common than separation during chewing. The ruptured cells release their contents, making them available for digestion. The proportion of nutrients made available will depend on the ratio of ruptured to intact cells, which increases as particle size decreases. Therefore, the smaller the particle the greater the proportion of nutrients available (Cassady et al., 2009; Ellis et al., 2004). This was clearly demonstrated by a study in which carbohydrate-rich foods (apple, potato, rice, and sweet corn) that were swallowed without chewing evoked a reduced postprandial glycemic response compared to when chewed (Read et al., 1986). It does not, however, apply to cooked legumes in which the cells have a tendency to separate. Leguminous cells may remain largely intact following mastication, leaving the intracellular starch protected from digestive enzymes (Noah et al., 1998).

### The stomach

Masticated foods may be swallowed as particulates or be re-formed into a bolus prior to swallowing. The bolus protects salivary amylase from acidic conditions in the stomach, thereby prolonging salivary digestion. If food is swallowed as particulates, any unprotected salivary amylase would normally be inhibited by the acidic pH, thereby ending salivary digestion (Fried et al., 1987; Rosenblum et al., 1988). In the stomach, the acidic pH and enzymic degradation of lipid and protein may help to soften the food structure. Although gastric juice is acidic (˜pH 2.0), digestion in the stomach generally occurs at a higher pH due to the buffering capacity of food. When food enters the stomach, the gastric pH will rise to approximate the original pH of the food when it was ingested, then gradually drop to pH 2.0 towards the end of gastric digestion (Malagelada et al., 1976). Mixing in the upper part of the stomach is gentle, and it may take up to one hour until gastric secretions penetrate to the centre of a bolus. In the lower stomach, further reductions in particle size are aided by the high shear motility. Liquids are digested faster than semisolids or cellular structures (Norton et al., 2007). Larger particles (>2 mm) are preferentially retained in the stomach in order to reduce particle size further, until eventually any remaining large particles are emptied through a ‘housekeeper-wave’ (Kong and Singh, 2008; Wickham et al., 2012). Although there may still be large numbers of intact cells, the acid environment will release some sugars. The soluble NSPs may also affect viscosity and mixing (McDougall et al., 1996). Therefore, the rate and extent to which the particle size of a food matrix is reduced during chewing and gastric digestion, is likely to influence, to some extent, the gastric emptying rate (Mourot et al., 1988; Pera et al., 2002).

### The small intestine

Chyme leaving the stomach is buffered by bicarbonate and mucin from the Brunner's glands and exposed to bile from the gallbladder and other digestive proteases, lipases, and amylolytic enzymes. Starch is hydrolyzed by pancreatic α-amylase to yield predominantly maltose, maltotriose, isomaltose, α-limit dextrins and a number of linear α-(1→4) linked polyglycan chains. These are further digested by disaccharidases, notably maltase-glucoamylase (MGAM), which hydrolyses maltose, β-limit dextrins and cyclodextrins, and sucrase-isomaltase, which hydrolyses sucrose and isomaltose. The resulting monosaccharides (i.e., glucose, galactose, and fructose) are then absorbed from the intestinal mucosa into the portal blood through GLUT2 transporters and sodium dependant transporters sodium-dependent glucose co-transporter (SGLT) (Kellett et al., 2005; Sim et al., 2008). Starch or starch hydrolysis products which escape digestion in the small intestine are termed “RS.” RS is classified into five types, with starch entrapped in the food matrix and therefore physically inaccessible being termed RS1, native (uncooked) starch granules RS2, retrograded starch formed after starch gelatinization RS3, chemically modified starch RS4 and starch capable of forming complexes between amylose and long branch chains of amylopectin with lipids RS5. The RS content of food is highly variable (i.e., cereals contain <3% RS whereas green bananas contain ˜75%) and depends on processing conditions (Andersson, 1992).

NSPs that contribute to increased viscosity have been suggested to reduce or delay the absorption of carbohydrates and fat in the upper part of the small intestine. The effect is due to impeded transport of nutrients to the absorbing surface (Brownlee, 2011) and/or delayed transport of digestive enzymes to their substrates (Schneeman, 1987), resulting in lower blood concentrations of glucose, insulin, and cholesterol. Other mechanisms have also been suggested. LM pectin has been shown to reduce the activity of amylase and lipase in duodenal fluid in vitro (Isaksson et al., 1982). The mechanism suggested was partly due to a decrease in pH caused by the addition of LM pectin. Furthermore, direct molecular interaction and adsorption has also been suggested as mechanisms in which NSPs decrease pancreatic enzyme activity (Dutta et al., 1985; Dunaif and Schneeman, 1981).

In vitro digestion of β-glucan solutions/dispersions (as simplistic models for cereal based drinks) have shown large effects on the solubility and aggregation state. It has been suggested that β-glucan “solutions” typically consist of both individually dissolved chains and primary aggregates built up of rather few polymer chains (Håkansson et al., 2012; Grimm et al., 1995). During digestion the gastric conditions cause either partial degradation and/or dissociation of aggregated structures (Ulmius et al., 2012b). Upon being exposed to small intestinal conditions, considerable reaggregation occurs. The study illustrates the need for adequate characterization and the problems that may arise when assumptions about functionality during digestion are drawn from the properties of the food before digestion.

### The colon (large intestine)

**Figure 4.**
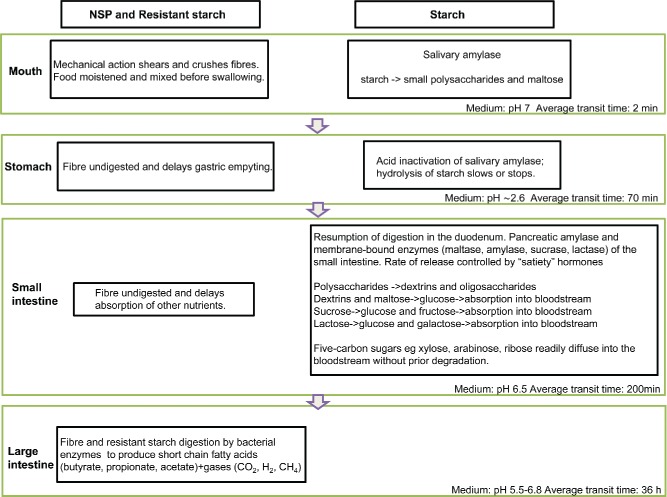
Schematic diagram of carbohydrate digestion through the digestive tract. It is recognized that transit times are not very reliable and we have therefore quoted average times. Some foods reside in the stomach for longer or shorter periods of time. In ileostomy subjects, the remaining part of the meal may reach the terminal ileum after more than four hours while the remaining parts of an almond meal came out of the stoma at 9–12 hours (Maurer et al., 2013).

Carbohydrates that have not been digested or absorbed in the upper GI tract enter the colon. They include the different forms of RS (physically entrapped, native, retrograded, or chemically modified starch) with about 80–90% of the ingested RS being fermented into SCFAs, particularly not only butyrate, but also propionate, acetate, isolvalerate, valerate, and isobutyrate (Cummings and Macfarlane, 1991; Asp et al., 1996). Butyrate is the major energy source for the colonic epithelial cells and uniquely has the ability to promote a normal phenotype in colonocytes by repairing damaged DNA (Le Leu et al., 2005; Toden et al., 2007; Topping et al., 2008). Propionate moderates hepatic lipid metabolism and acetate is metabolized by peripheral tissues (Wong et al., 2006). However, the vast majority of carbohydrate entering the colon is NSP (Mishra and Monro, 2012). Bacterial enzymes in the colon ferment NSPs (cellulose, hemicelluloses, and pectin) in the anaerobic environment of the colon producing SCFAs, the yields and ratios depending upon which polysaccharide is fermented. Up to 10% of dietary energy is provided by the metabolism of the SCFAs produced (Mishra et al., 2012; Mishra and Monro, 2012). Pectin yields more acetate (Englyst and Hudson, 1987), β-glucan more acetate and propionate than butyrate (Hughes et al., 2008), and arabinoxylan more acetate and butyrate than propionate (Hughes et al., 2007). The size of the NSP as well as the degree of cross-linking to phenolic compounds (lignin) will affect the degree and rate of fermentation, as will solubility. The oligosaccharides fructans, raffinose, lactose, and stachyose also pass into the colon and are readily fermented to SCFAs (Cummings and Macfarlane, 1991). The prebiotic effects of NSPs, fructans, lactulose, and gluco-oligosaccharides in promoting the populations of bacteria that produce SCFAs (notably) Bifidobacteria and Lactobacilli (Manning and Gibson, 2004) are now reasonably well accepted. Cellulose is fermented very slowly and is considered to be the main component of dietary fiber that contributes to fecal bulk (Cummings, 1982; Monro and Mishra, 2010). Fig. 4 is a schematic diagram of carbohydrate digestion through the digestive tract. It is probable that phenolic acids bound to cereal arabinoxylan (principally ferulic acid) are also released by fermentation in the colon allowing their absorption into the bloodstream (Vitaglione et al, 2008).

## Health impacts

Carbohydrates are important for glucose homeostasis, oxidative metabolism, and gastrointestinal function. However, they differ greatly in their potential to exert these effects, with their physical and chemical properties affecting the rates at which they are hydrolyzed with impacts on digestive physiology, gut hormone signaling and postprandial metabolism. Starch is particularly important as it forms a major part of the carbohydrate supply in the human diet. Researchers have therefore investigated the potential selection and/or manipulation of specific carbohydrates for therapeutic use, particularly their potential roles in the management and prevention of diabetes, obesity, cardiovascular disease, and metabolic syndrome (Wolever et al., 1992; Wolever, 2000; Jenkins et al., 2002).

### Energy balance/satiety

Current guidelines recommend that 55% of total dietary energy should be sourced from carbohydrates (FAO/WHO 1998). Conventionally (on the basis of Atwater factors), 1 g of carbohydrate was assumed to provide 17 kJ/g (4 kcal/g); however, as carbohydrates vary greatly in their digestibility and metabolic potential, this can no longer be accepted as a reliable figure (Zou et al., 2007). Depending on the site, rate, and extent of carbohydrate digestion, metabolic energy yield is likely to vary. Fully fermentable RS has been estimated to contribute ˜8.8 kJ/g (2 kcal/g), whereas glucose contributes 17 kJ/g.

The role of carbohydrates in obesity is controversial. It is also important to distinguish between different categories of carbohydrates, as sugar, starch, RS, and NSPs may exert different physiological effects. For example, increased intake of sugar-sweetened beverages has been associated with an increased risk of developing obesity, probably because these sugars do not induce satiety to the same extent as more complex carbohydrates (van Dam and Seidell, 2007). On the other hand, satiety-promoting carbohydrates may potentially be used to aid weight loss (Norton et al., 2007). Rebello et al. (2013) recently reviewed the role of carbohydrates on satiety and food intake. Three possible mechanisms were explored; hormonal effects on satiety mediated by insulin and GI hormones; the intrinsic properties of carbohydrates that relate to bulking and possible viscosity effects, and bulking and possible viscosity effects, and fermentation of undigested carbohydrates to SCFAs by colonic microbiota. Foods with low glycemic index, which may contain more RS, have been shown to reduce weight gain in a similar way to NSPs; however, there is currently insufficient evidence to demonstrate that such foods reduce the risk of developing obesity or aid weight loss (van Dam and Seidell, 2007).

The rate of digestion also determines how sustained the supply of glucose will be as digestion continues, and therefore, how prolonged its contribution to delaying the urge to eat again will be (Mishra and Monro, 2012). Termination of the period of satiety coincides with the resurgence of the feeling of hunger, leading to consumption of the next meal with a consequent resumption of the food intake cycle (Bornet et al., 2007). One proposed mechanism to affect weight loss is through increased satiety (Singh and Sarkar, 2011; Kendall et al., 2010). Studies have shown that low GI foods (see below), which are also rich in fiber, lead to prolonged satiety and reductions in food intake (Ludwig, 2002; Abete et al., 2011).

### Glycemia

The glycemic index (GI) was introduced by Jenkins et al. in 1981 as a means of documenting the glycemic response to different carbohydrate foods. GI refers to the glycemic effect of available carbohydrate (usually 50 g) in a food relative to the effect of an equal amount of available carbohydrate (usually from white bread or glucose), which is defined as having a GI of 100. Since then there have been some improvements in the methodology used to determine GI resulting in revised GI tables (Foster-Powell et al., 2002). Foods classified as high GI include refined-grain products, white bread and potato, whereas low GI foods include whole-grain products, legumes, and fruits.

The rate and extent of starch digestion in the intestinal lumen plays a crucial role in regulating the rise in post-prandial blood glucose and insulin concentrations (Warren et al., 2011). The glycemic response to a food is particularly important for people with abnormalities in blood glucose regulation, notably those with type 2 diabetes or metabolic syndrome (Whitney et al., 1990, Sizer and Whitney, 2000). Currently, it is estimated that type 2 diabetes affects more than 2.9 million people in the United Kingdom (2012) (http://www.diabetes.org.uk) and its prevalence is increasing rapidly. This disease is characterized by insulin resistance, and consequently the mechanisms involved in maintenance of glucose homeostasis are impaired. In healthy individuals, the postprandial rise in plasma glucose concentration is counteracted predominantly by the hormone insulin, which stimulates the increased utilization and storage of glucose by glycolytic tissues, thereby reducing circulating glucose concentration to maintain homeostasis. A large number of studies have shown the efficacy of low GI foods in the dietary management and prevention of obesity, diabetes, and cardiovascular disease (Jenkins et al., 2002; Wolever et al., 1992; Wolever, 2000; Brand et al., 1991).

Foods with a low GI are digested slowly and do not cause large fluctuations in postprandial glycemia. This indicates that circulating glucose levels are effectively reduced, while glucose uptake in the small intestine continues (Jenkins et al., 2002). On the basis of this mechanism, low GI foods may be beneficial in the prevention and management of diabetes, obesity, and cardiovascular disease (Wolever et al., 1992; Wolever, 2000; Jenkins, et al., 2002). However, GI remains controversial (for use as a food label) as the amount of food consumed is not taken into account and can be challenging to measure (Aziz et al., 2013).

There are many factors that influence the postprandial glycemic and insulinemic response to food. These include the digestibility of the starch; the natural structure of the food matrix (Liljeberg et al., 1992; Jarvi et al., 1995; Singh et al., 2010; Kristensen et al., 2010); viscosity of the digesta (Kaur et al., 2007); interactions of the starch with protein in the food (Jenkins et al., 1987); the amount and types of fat (Henry et al., 2008; Clegg et al., 2012); sugars and NSPs (Hardena et al., 2012); and the presence of other constituents, such as α-amylase inhibitors, phytate, and polyphenols which may impair starch digestibility (Savelkoul et al., 1992; Dhital et al., 2010; Garcia-Alonso and Goni, 2000). Also, the textural and rheological characteristics of food may be affected by food processing and influence the digestibility of starch.

### Colonic health

The extent of digestion during transit through the upper GI tract is important as it determines the proportion of starch that is available to the colon for fermentation (Fuentes-Zaragoza et al., 2010; Björk et al., 2012), and may also play a role in appetite control through the colonic brake feedback mechanism (Brownlee, 2011). Undigested food residues, including both cell walls and the carbohydrates and other nutrients that they have protected or escape from digestion, are now recognized as a valuable feedstock for the colonic ecosystem (Buttriss and Stokes, 2008).

The recommended daily intake of dietary fiber in the United Kingdom is 18 g per day (Department of Health, 1999), although the average actual intake is only about 13 g per day (Buttriss and Stokes, 2008). Recommended intakes are generally higher in other European countries, being about 25–40 g a day. Most of this is NSPs, as the daily intake of RS in European countries is estimated as 4.1g/d RS compared with 15–20g/d of fermentable fiber (Cummings, 1983).

RS has similar attributes to NSPs in that it is fermented by colonic micro-organisms into SCFAs. Both NSPs and RS increase fecal bulk, by increasing the volume of bacteria. However, the effect of RS is minor in comparison with the effects of NSPs in bran, fruit, and vegetables (Cummings et al., 2004). This may be due, to the lower amount of RS delivered to the colon compared to NSPs in Western diets (Lobley et al., 2013). There may be differences between different types of NSP and RS, and further research is required to understand the mechanism(s).

As discussed earlier, NSPs and RS are fermented in the colon to produce SCFAs, and butyrate has received the most attention as it has beneficial effects on colonocytes, suggesting a mechanism for beneficial effects on colorectal cancer (Topping et al., 2003; Le Leu et al., 2005; Toden et al., 2007). The fermentation of RS results in the production of higher levels of butyrate compared to NSPs (Topping and Clifton, 2001; Topping et al., 2008), while fermentation of arabinoxylan results in greater production of butyrate compared to fermentation of β-glucan (Hughes et al., 2007; 2008).

Propionate is absorbed and metabolized aerobically in the liver while acetate passes via the liver into the blood from where it is used as an energy source. It is increasingly recognized that the products of colonic fermentation influence the body as a whole, through effects on the immune system mediated by the colonic epithelium, and through neuronal and hormonal feedback from the colon to upstream regions of the digestive tract (Wikoff et al., 2009). Propionic acid in particular, may play a direct role in blood glucose control by suppressing the release of plasma triacylglycerols, which contribute to insulin resistance (see above). Colonic fermentation also appears to have indirect effects on hormones from the pancreas and adipose tissue that are involved in the regulation of energy metabolism (Nilsson et al., 2008). Indeed, it has been suggested that obesity is associated with a colonic microbiota that is more effective at scavenging energy from undigested food polysaccharides than the microbiota from lean individuals (Taunbrugh et al., 2006).

### Vascular function

NSPs have also been suggested to reduce the reabsorption of bile acids (sometimes explained as “binding to bile acids” as demonstrated in vitro (Kritchevsky and Story, 1974; Brownlee, 2011), resulting in synthesis of new bile acids from cholesterol and hence, reducing blood cholesterol levels (Theuwissen and Mensink, 2008). The increased viscosity caused by the presence of NSPs is known to contribute to the lowering of fasting blood cholesterol (Ellis et al., 1995). Additions of liquid (dilution) to the food matrix during digestion, which can be quite considerable depending on the food, can also be expected to reduce the viscosity. Therefore, it is uncertain what the actual viscosity of a given food matrix is in the small intestine. Ferulic acid released by the fermentation of cereal arabinoxylan in the colon may have antiproliferative effects on colonocytes, reducing the risk of colon cancer (Janicke et al, 2011), and have antihypertensive effects on vascular function (Suzuki et al, 2007; Alam et al, 2013).

## Conclusion

The carbohydrate components of the human diet are derived almost exclusively from plant sources and play crucial roles in food processing and in diet and health. Although widely regarded as primarily sources of energy they also have other impacts on diet and health, particularly the cell wall polysaccharides, which are the major components of dietary fiber. It is becoming clear that both dietary fiber and resistant forms of starch play a positive role in reducing risk factors for chronic diseases, including cardiovascular disease and certain types of cancer. These benefits could be exploited by crop and food scientists to develop new foods to combat the epidemic increases in diet-related disease which are occurring in both developed countries and rapidly expanding economies such as China and India.
